# Phytochemical Screening by HRLC–MS/MS (Q-TOF) and Antioxidant and Anti-Inflammatory Properties of *Thottea sivarajanii* Leaf Extract

**DOI:** 10.3390/ph18121794

**Published:** 2025-11-25

**Authors:** Pooja Mohan Padmalayam, Aswathi Moothakoottil Kuttithodi, Alby Tom, Joice Tom Job, Satheesh George, Arunaksharan Narayanankutty

**Affiliations:** 1 Division of Cell and Molecular Biology, PG and Research Department of Zoology, St. Joseph’s College (Autonomous), Devagiri, Affiliated to University of Calicut, Calicut 673008, Kerala, India; poojamohan@devagiricollege.org (P.M.P.); aswathimk@devagiricollege.org (A.M.K.); albytom@devagiricollege.org (A.T.); joicetomj@gmail.com (J.T.J.); 2 Department of Botany, St. Joseph’s College (Autonomous), Devagiri, Affiliated to University of Calicut, Calicut 673008, Kerala, India

**Keywords:** *Thottea sivarajanii*, Aristolochiaceae, Ethnomedicine, LC-MS analysis, pharmacology, natural product

## Abstract

**Background**: Numerous degenerative diseases are brought on by inflammation and oxidative stress. Metabolites from plants contain anti-inflammatory and antioxidant properties. Indigenous and understudied, *Thottea sivarajanii* is a significant ethnobotanical herb. It is native to the Western Ghats and belongs to the Aristolochiaceae family. **Objectives**: The current study investigated the antioxidant and anti-inflammatory properties of *T. sivarajanii* leaf methanol extract (TSL) and the insights provided by phytochemical analysis. **Methods**: The HRLC–MS/MS (Q-TOF) study is used for the phytochemical analysis. The antioxidant efficacy is evaluated in terms of DPPH and ABTS radical scavenging, and reducing power (FRAP assay). In vitro anti-inflammatory efficacy was evaluated on RAW 264.7 cells challenged with lipopolysaccharide (LPS). **Result**: The HRLC–MS/MS (Q-TOF) study indicated the presence of bioactive molecules such as ursolic acid, Daidzein 4’,7-diglucoside, Calophyllin B, and Berbamine, etc. The results showed in vitro antioxidant capacity in DPPH, and ABTS, radical scavenging, and ferric-reducing activities with respective IC_50_ and EC_50_ values of 184.5 ± 2.4, 24.15 ± 0.13, and 4.94 ± 0.32 µg/mL, respectively. LPS significantly stimulated the production of IL-1β, IL-6, and TNF-α in RAW 264.7 cells (*p* < 0.001). Treatment with TSL reduced levels of IL-1β and IL-6 from 776.1 ± 11.4 and 1678.1 ± 12.4 to 195.4 ± 9.2 and 465.4 ± 11.8 pg/mg protein. It also reduced NO levels from 91.4 ± 1.3 to 30.8 ± 1.7 µM/mg protein while reducing TNF-α levels from 2041.2 ± 15.1 to 1037.5 ± 15.4 pg/mg protein. **Conclusions**: This work contributes to the growing evidence supporting the pharmacological importance of the underexplored *Thottea sivarajanii*, highlighting this species as a promising candidate for natural antioxidant and anti-inflammatory agents.

## 1. Introduction

Reactive oxygen species (ROS) are regularly generated by living cells as a part of cellular metabolism. ROS is important in the immune system for the eradication of pathogens [1]. The bodily amount of oxygen or nitrogen-free radicals is called oxidative eustress [2], which is engaged in numerous biochemical events such as carboxylation, hydroxylation, and signaling pathways, including NF-KB, MAPK cascade, phosphoinositide-3-kinase, and Nrf2 cascades [2,3]. Short-term oxidative stress may prevent aging by promoting mitohormesis [4]. The antioxidants in cells balance out the adverse effects of ROS [5]. Oxidative stress results from an imbalance between the production of ROS and the ability of cells to scavenge the same [6]. ROS can damage cellular components like membranes, lipids, proteins, and lipoproteins [7]. The interactions between ROS and DNA lead to the base modifications, and DNA-protein crosslinking, oxidation of deoxyribose, strand breakage, and nucleotide removal [8]. The antioxidant defense system consists of both endogenous (enzymatic and non-enzymatic) and exogenous antioxidants [9]. Antioxidants like glutathione, vitamin C, and vitamin E scavenge ROS directly, whereas superoxide dismutase (SOD), catalase, and glutathione peroxidase are enzymatic antioxidants [10]. The transcription factor Nrf2 is the vital regulator of the genes that are involved in the antioxidant defense mechanism [11].

Inflammation is the physiological response to fluctuations in tissue homeostasis that involves both innate and adaptive immunity [12]. Interleukins are cytokines that regulate the immune system’s inflammatory reactions. IL-1b and IL-6 are pro-inflammatory interleukins required to initiate an immune response. The interleukins, IL-10 and TNF-β, are anti-inflammatory interleukins that play an important role in limiting inflammation and reducing tissue damage by stimulating the development and activation of regulatory immune cells [13]. Increased release of anti-inflammatory cytokines may cause immunological suppression and diminished pathogen clearance. Uncontrolled production of pro-inflammatory interleukins triggers tissue damage and sepsis [14]. Both inflammation and oxidative stress are interrelated pathways in the sense that inflammatory cells produce ROS at the site of inflammation, while ROS/reactive oxygen and nitrogen species (RONs) increase pro-inflammatory gene expression by participating in signaling cascades [15]. Extended oxidative stress and inflammation may cause diabetes, cancer, pulmonary disease, and inflammatory bowel disease [16], cardiovascular and neurological diseases (Alzheimer’s disease, Parkinson’s disease) [16,17], aging [17], depression, schizophrenia, and bipolar disorder [3] and male [18] and female [19] fertility issues.

Folk medicine comprises ancient healing practices using plant, animal, and mineral remedies, passed down orally through generations. It is rooted in culture and community beliefs [20]. Bioactive metabolites exhibit a wide range of pharmacological actions and are classified as terpenoids, phenolics, flavonoids, alkaloids, and glycoside chemicals based on their chemical structure and functional groups [21]. They frequently interact with proteins, bio-membranes, and nucleic acids [22]. These activities could be potential treatments for a variety of diseases, and unlocking their activities may shed light on the treatment of many severe diseases [23]. Plant metabolites have antioxidant activity [24], anti-inflammatory activity, cardiovascular protection, anti-cancer activity, and anti-nociceptive activity [25]. Their therapeutic potential is particularly significant considering the adverse effects associated with many allopathic drugs, including facial swelling, rashes, headaches, inflammation, and the development of drug resistance [26]. There has been an upsurge in global demand for traditional remedies [27].

The Aristolochiaceae family is known for its therapeutic properties and is commonly utilized in Chinese medicine to treat various maladies [28]. Aristolochiaceae is an important source of various bioactive compounds; this includes various polyphenols, alkaloids, terpenoids, and flavonoids [28]. The genus *Thottea* is rarely investigated for scientific validation of its pharmacological effects. *Thottea siliquosa* has been extensively studied for its pharmacological characteristics. *T. siliquosa* root has promising anti-inflammatory activity [29] and antibacterial and antioxidative activities [30,31]. It is a natural source of chemicals with anti-cancer and cytotoxic potential [31,32]. Also, the leaves reduce lipopolysaccharide-induced cytokine production [33] and protect against ethyl methyl sulfonate-induced genotoxicity [33]. In mice, *T. siliquosa* leaves prevent carrageenan and formalin-induced paw edema [34]. *T. barberi* roots contain organic chemicals that can replace synthetic aspirin [35]. *T. ponmudiana* have antibacterial activity [36,37], whereas the *T. tomentosa* demonstrate antioxidant and antimicrobial activity [38]. *T. sivarajanii* is endemic to India and was first documented in the Western Ghats [39]. This work intends to validate the pharmacological capabilities of the underexplored plant *T. sivarajanii* by assessing its in vitro antioxidant and anti-inflammatory activities, based on phytochemical analysis of its leaf methanolic extract.

## 2. Results

### 2.1. Phytochemical Characterization

**Figure 1:**
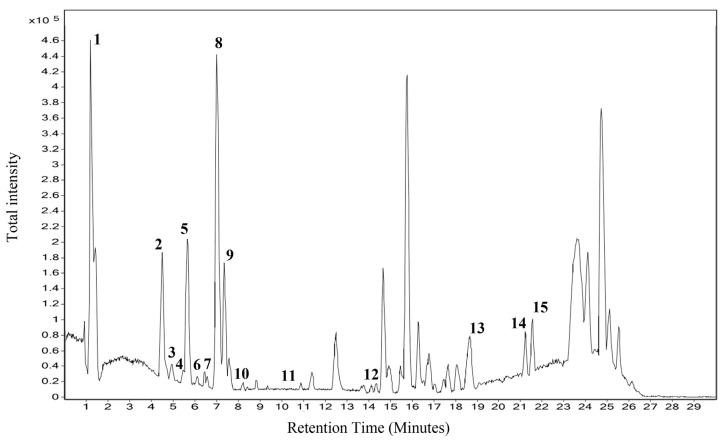
LC–MS total ionization chromatogram of *Thottea sivarajanii* leaf methanol extract.

The methanolic extract of *T. sivarajanii* leaves had a total phenolic content of 34.26 ± 2.60 GAE/g dry leaf, and the flavonoid extract measured 40.03 ± 1.27 QE/g of dry leaves. The chemical constituents of the TSL were subjected to LC–MS analysis, and the results obtained were compared with the spectral library (Figure 1). The extract contains ursolic acid, Daidzein 4’,7-diglucoside, Calophyllin B, and Berbamine, etc., (Table 1).

### 2.2. In Vitro Antioxidant Capacity

**Figure 2:**
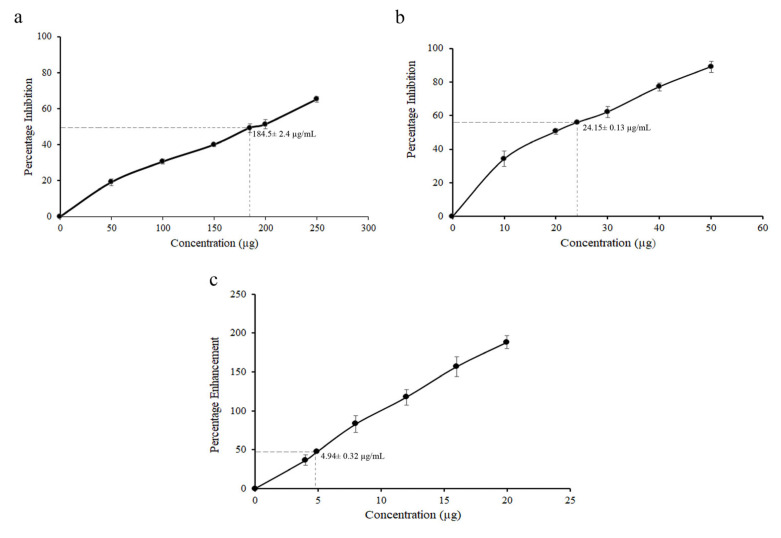
Antioxidant capacity of TSL in different assays. (**a**) DPPH assay, (**b**) ABTS assay, and (**c**) FRAP assay.

The antioxidant capacity of the TSL was assessed using DPPH, ABTS, and FRAP tests (Figure 2a–c). The IC_50_ values for extract DPPH and ABTS scavenging were 184 ± 2.4 and 24.15 ± 0.13 µg/mL, respectively. The FRAP assay yielded an EC_50_ value of 4.94 ± 0.32 µg/mL (Table 2).

### 2.3. Anti-Inflammatory Activity in RAW 264.7 Cells

**Figure 3:**
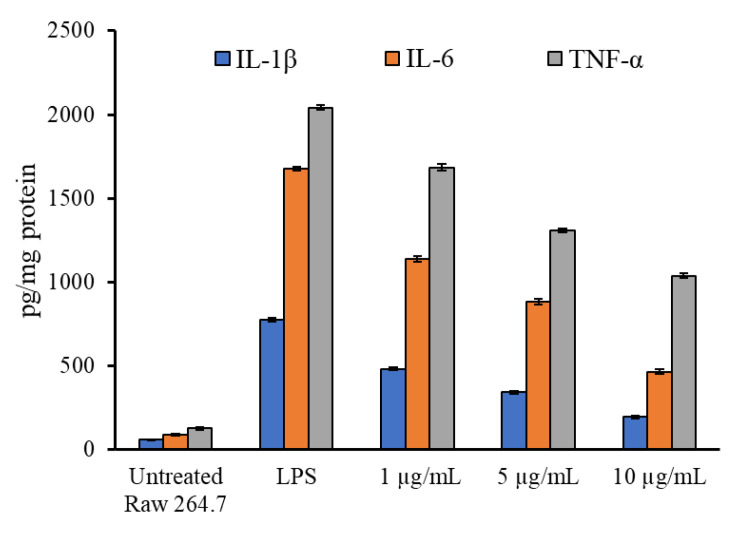
Anti-inflammatory activity of *Thottea sivarajanii* leaf methanolic extract against lipopolysaccharide-induced IL-1β, IL-6, and TNF-α release in RAW 264.7 cells.

**Figure 4:**
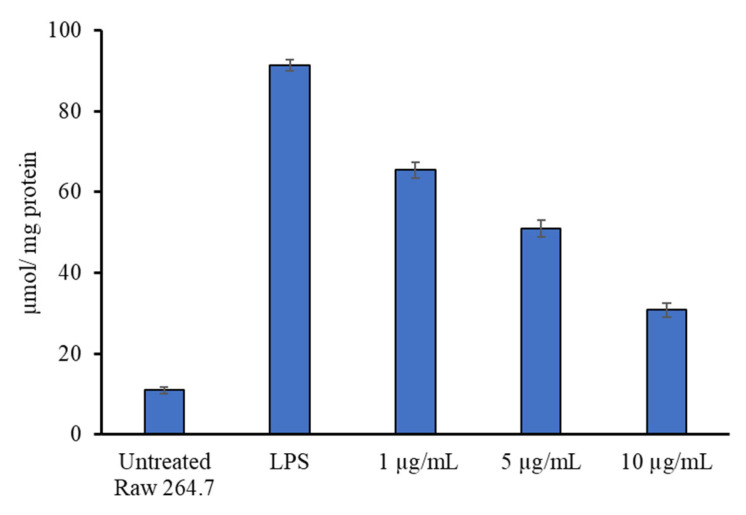
Anti-inflammatory activity of *Thottea sivarajanii* leaf methanolic extract against lipopolysaccharide-induced nitric oxide production in RAW 264.7 cells.

The biologically safer concentrations of the *T. sivarajanii* leaf extract were determined and used for the anti-inflammatory activity analysis (Supplementary Figure S1). RAW 264.7 cells treated with LPSs produced more pro-inflammatory cytokines and nitric oxide radicals. Figure 3 highlights the anti-inflammatory effects of plant extract in LPS-primed macrophage cells. Pro-inflammatory cytokines were dramatically enhanced in LPS-stimulated macrophages. However, pretreatment with the plant at 10 µg/mL dosages significantly inhibited IL-1β and IL-6 release (Figure 3). In a dose-dependent way, the extract reduced the levels of nitric oxide generation in LPS-activated macrophage RAW 264.7 cells. The amount of NO produced by LPS-activated RAW 264.7 cells was 91.4 ± 1.3 µmol/mg protein. After the LPS-activated cells were pre-treated with 1 µg/mL and 10 µg/mL of extract, it dropped to 65.4 ± 2.0 and 30.8± 1.7 µmol/mg protein, respectively (Figure 4). The percentage changed in the levels of cytokines and NO production in *T. sivarajanii* leaf extract with respect to the LPS alone-treated macrophages (Supplementary Table S1). At 1.0 µg/mL, the extract suppressed IL-1β, IL-6, TNF-α, and NO by 37.8 ± 0.1%, 32.04 ± 0.1%, 17.53 ± 0.1%, and 28.45 ± 0.1%, respectively. Increasing the concentration to 5.0 µg/mL enhanced the inhibitory effect, showing 56.0 ± 0.2% reduction in IL-1β, 47.43 ± 0.2% in IL-6, 35.90 ± 0.2% in TNF-α, and 44.31 ± 0.2% in NO. The highest tested concentration, 10.0 µg/mL, produced the most pronounced suppression, decreasing IL-1β, IL-6, TNF-α, and NO by 74.8 ± 0.2%, 72.27 ± 0.2%, 49.17 ± 0.2%, and 66.30 ± 0.2%, respectively.

## 3. Discussion

Herbal drugs are widely accepted in communities due to their low risk of adverse effects. The knowledge of medicinal plants and their phytochemical study sheds light on the role of secondary metabolites as medications. Ethnobotanically significant plants possess antioxidant qualities because of their phytochemical makeup. Flavonoids and phenols are the primary constituents. The structure of these elements allows them to scavenge the free radicals [24]. Polyphenols are a major component in plant extracts, and contribute to antioxidant action because they contain hydroxyl groups [40]. They have both antioxidant and anti-inflammatory activities, such as decreasing ROS formation and increasing cell antioxidant defense, as well as lowering pro-inflammatory enzymes [41].

In the current investigation, *T. sivarajanii* showed substantial antioxidant capabilities. A compound’s capacity to donate electrons is its reducing power; therefore, in FRAP, the antioxidant potential of a compound can be indicated by its reducing capacity [35]. In comparison with the FRAP capacity of another species of the same genus, *T. siliquosa* (41.1 ± 6.2 µg/mL), the ferric reducing potential of *T. sivarajanii* leaf extract (4.94 ± 0.32 µg/mL) showed an eight-fold activity. The LC–MS analysis verified the occurrence of bioactive compounds like ursolic acid, Daidzein-4,7 diglucoside, and syringaresinolO-beta-D-glucoside, etc. Ursolic acid exhibits antioxidant, anti-inflammatory, anti-cancer, [42,43] and anti-diabetic properties [44]. Through the IER3/Nrf2-signaling pathway and the Nrf2/antioxidant response element-signaling pathway, ursolic acid can effectively reduce oxidative stress [45].

Herbal medicine components have shown the capacity to modulate inflammatory responses in the body, either directly or indirectly [46]. Lowering the levels of TNF-α, IL-6, and IL-1β can prevent the development and progression of inflammatory and degenerative diseases [47]. These cytokines drive the gene expression of chemokines, downstream inflammatory mediators, and adhesion molecules that accelerate the inflammatory response and foster cell death through NF-κB and JAK/STAT pathways [48]. These pathways can be diminished by suppressing the synthesis of cytokines or restricting their binding with receptors and thereby minimizing damage to organs induced by chronic inflammation [47].

In biological systems, the isoflavone daizein glycosides have antioxidant, anti-inflammatory, and anti-cancer properties [49]. NF-κB is modulated by Daidzein binding, which results in a drop in TNF-α, COX-2, iNOS, and NLRP3, and subsequently a decrease in IL-1β and IL-18 activation [50]. Also, Daidzein and its derivatives can prevent NF-κB p65 and ERK1/2 from being phosphorylated and thereby lower IL-6 production [51]. Berbamine (BER) is a bisbenzylisoquinoline alkaloid that has been identified in *Berberis amurensis* [52] BER suppressed the activation of NF-κB and MAPK (JNK and ERK1/2) signaling pathways, thereby dramatically reducing the production of inflammatory factors by LPS-stimulated macrophages [53]. The anti-inflammatory effect is also explained by the presence of calophyllin B [54] and syringaresinol O-beta-D-glucoside [55]. Inflammation generates ROS and nitric oxide (NO), which induce lipid oxidation and peroxidation damage [56]. NO can affect a wide range of elements of the inflammatory cascade [57].The pretreatment of RAW 264.7 cells with varied concentrations of TSL dramatically reduced interleukin and NO production.

## 4. Materials and Methods

### 4.1. Plant Collection and Extraction

The plants were collected in May 2024 from Kakkayam forest (11.553819° N, 75.920339° E), Malabar Wildlife Sanctuary, Kozhikode, Kerala. The plant was identified by taxonomist Dr. Satheesh George, St. Joseph’s College (Autonomous), Devagiri, and voucher specimen (Voucher no. 7503) was deposited in the Herbarium facility of St. Joseph’s College (Autonomous), Devagiri, Kerala. After gathering the plants, the leaves were dried in the shade and ground into a coarse powder. In a Soxhlet extractor, 10 g of powder was extracted for 6–8 h using methanol as the solvent. The temperature employed during Soxhlet extraction remained below the boiling point of methanol, thereby minimizing thermal degradation. The extract was then dried in the presence of nitrogen to completely remove methanol. The obtained sample represents the crude methanolic extract of the plant, without prior defatting.

### 4.2. Cell Lines and Maintenance

The mouse (*Mus musculus*) macrophage cell line RAW 264.7 (TIB-71-ATCC) was used as the model in the study. The cells were procured from the National Cell Repository, National Centre for Cell Science, Pune, India. The cells were analyzed for mycoplasma contamination and found to be non-contaminated using PCR kits (Himedia, Banglore, India). The cells were authenticated and found to be free from mycoplasma contamination. The cells were maintained under standard atmospheric conditions of 5% CO_2_ in an incubator cultured in Dulbecco’s Modified Eagle’s Medium (DMEM) media (Cat. No. 10313021, Gibco, Grand Island, New York, NY, USA) supplemented with 10% fetal bovine serum, 4.5 g/L glucose, sodium bicarbonate (3.7 g/L), 1 mM sodium pyruvate, 4 mM L-glutamine, and 1% penicillin and streptomycin antibiotics (each 5000 U/mL).

### 4.3. Total Phenol Content

Working standards for gallic acid can be obtained by diluting the 1.0 mg·mL^−1^ stock to 20, 40, 60, 80, and 100 µg·mL^−1^. Take 0.50 mL of the standard and 0.50 mL of the suitably diluted sample in a test tube. Pour in 2 mL of Folin–Ciocalteu reagent. Let the mixture remain at room temperature for 6 min. Add 4 mL of Na_2_CO_3_ at 7%. To make the total volume of the reaction mixture 10 mL, add 3.5 mL of distilled water. Stir well. For half an hour, let the reaction mixture sit at room temperature in the dark. Compare the absorbance at 550 nm to a blank for the reagent [58]. Run each standard and sample three times. Add a control and a reagent blank. Create a calibration curve by plotting absorbance (y) against concentration (x, µg·mL^−1^) using gallic acid standards.

### 4.4. Total Flavonoid Content

The aluminum chloride reaction [59] was used to determine flavonoid content and final concentration expressed in quercetin equivalents. A stock solution of quercetin was prepared by dissolving 10 mg of quercetin in 10 mL of methanol. Working standard solutions (10, 20, 40, 60, 80, and 100 µg/mL) were prepared by serial dilution of the stock solution with methanol. To each tube containing the standards, samples, and blank, 300 µL of 5% NaNO_2_ solution was added, mixed well, and incubated for 6 min. Subsequently, 300 µL of 10% AlCl_3_ solution was added and the mixture was allowed to stand for 5 min. Then, 2 mL of 1 N NaOH was added, and distilled water was added to make the final volume 5 mL. The reaction mixtures were incubated for 15 min at room temperature in the dark, and the absorbance was measured at 550 nm. Run each standard and sample three times. Add a solvent control and a reagent blank. Create a calibration curve by plotting absorbance (y) against concentration (x, µg·mL^−1^) using quercetin standards.

### 4.5. HRLC–MS/MS (Q-TOF) Analysis

The analysis was performed using the method ESI_+VE_MS/MS. The HiP Sampler, (G4226A, Agilent Technologies, Santa Clara, CA, USA), Binary Pump (G4220B, Agilent Technologies, Santa Clara, USA), Column Component (G1316C, Agilent Technologies, Santa Clara, USA), DAD (G4212B, Agilent Technologies, Santa Clara, USA) and Q-TOF mass spectrometer were used for this analysis. The G6550A variant was equipped with a TOF/Q-TOF mass spectrometer. Dual AJS ESIs were employed as ion sources. Auto MS2 mode was utilized for acquisition, scanning the 120–1200 *m*/*z* range with an MS/MS Scan Rate (spectra/sec) of 1. Chromatographic separation was carried out using a Hypersil GOLD C18 column (100 × 2.1 mm, 3 µm), providing high-resolution separation of analytes. The mobile phase consists of two solvents: Solvent A, which is water with 0.1% formic acid, often used as a hydrophilic solvent, and Solvent B, acetonitrile, which is a more hydrophobic solvent. In this method, Solvent A was specifically 0.1% formic acid in Milli-Q water, while Solvent B consisted of acetonitrile, as used in the LC elution program. The chromatographic separation employs a linear gradient, transitioning from 5% B to 95% B over 30 min, which increases the solvent strength over time and helps elute more hydrophobic compounds later in the run. The flow rate is maintained at 0.300 mL/min to ensure a constant movement of the mobile phase through the column. The column temperature is set at 40°C to maintain consistent and efficient separation throughout the process. A 5.00 µL sample volume was injected into the LC system, with the injection mode including a needle wash step to prevent carryover between samples. The MS/MS scan rate was set to 1.00 spectra per second, allowing for rapid data acquisition. The isolation width for precursor ion selection is set to a medium value of approximately 4 amu. Precursor ions are selected for fragmentation based on their abundance and other criteria. Active exclusion prevents repeated fragmentation of the same ions, enabling the analysis of a broader range of compounds. The diode array detector records UV–Vis absorbance at multiple wavelengths, providing complementary information to the mass spectra. The Q-TOF acquires full-scan mass spectra to identify the *m*/*z* values of all ions present in the sample. Fragmentation spectra are then acquired for selected precursor ions, providing structural information to aid in metabolite identification and characterization.

### 4.6. In Vitro Antioxidant Studies

#### 4.6.1. DPPH

DPPH stock solution was made by mixing 2.02 mg of DPPH in 30 mL of methanol. Methanol was added to the DPPH solution until its absorbance at 517 nm was 1. A measurement of 150 mL of DPPH methanolic solution was mixed with varying concentrations of extract. After giving the mixture a good shake, it is left at room temperature for half an hour in the dark. The absorbance was taken at 517 nm. Its ability to neutralize or inhibit free radicals is measured in terms of IC_50_ [60].

#### 4.6.2. ABTS

An equivalent volume of a 2.45 mM potassium persulfate solution and a 7 mM ABTS stock solution were combined to create the ABTS solution. After that, the combination was kept for 12 to 16 h at room temperature in a dark place. Methanol was added to the ABTS solution until its absorbance at 734 nm was 0.7 ± 0.002. After mixing 2 mL of solution with varying concentrations of the sample, it is incubated for seven minutes in the dark. Radical scavenging activity was measured in terms of IC_50_ [61].

#### 4.6.3. FRAP

In order to create the FRAP reagent, 300 mM acetate buffer, 10 mM TPTZ solution in 40 mM HCl, and 20 mM FeCl_3_ are combined in a 10:1:1 ratio. Freshly made FRAP reagent was combined with various sample concentrations, and the mixture was incubated for 30 min at room temperature. Absorbance is measured at 593 nm. Antioxidant capacity is measured in terms of its ability to reduce Fe^3+^ into Fe^2+^, EC_50_ [62].

The values of in vitro studies are represented as mean ± SD of triplicate determinations with ascorbic acid run as standard.

### 4.7. Anti-Inflammatory Studies in RAW 264.7 Cells

In a 24-well plate with complete DMEM media, the RAW 264.7 cells were allowed to adhere (1 × 10^7^ cells/mL) for 24 h. The complete growth media were replaced with DMEM media containing the different concentrations (1.0, 5.0, and 10 µg/mL) of *T. sivarajanii* extract and incubated for another 24 h as pretreatment. The cells were then exposed for another 24 h with 1 µg/mL lipopolysaccharide in fresh DMEM media. At the end of incubation, cells were harvested and media were collected for further analysis. PeproTech ELISA kits (Rocky Hill, CT, USA) were utilized to measure the secreted levels of tumor necrosis factor-α and the protein expression of cytokines such as interleukin-1β and interleukin-6 under the commercially recommended protocols. The cells were lysed using the freeze–thaw method and centrifuged at 10,000× *g* for 15 min to yield the supernatant. The amount of nitric oxide in the cell lysate was measured using the Griess reaction method [63].

## 5. Conclusions

The *T. sivarajanii* leaf methanolic extract has anti-inflammatory and antioxidant effects. It demonstrated free radical scavenging ability and inhibition of pro-inflammatory mediators such as interleukins and cytokines. These results indicate that *T. sivarajanii* leaves contain bioactive compounds that are able to modulate oxidative stress and inflammatory pathways. Numerous chronic diseases are caused by prolonged inflammation and oxidative damage. Given the increasing demand for alternatives to regulate various types of prooxidative and inflammatory processes, using herbal products with antioxidant and anti-inflammatory components can be a smart choice. However, further investigations are required to support its therapeutic applicability.

## Figures and Tables

**Table 1:** Compounds identified through LC–MS analysis of *T. sivarajanii* leaf methanolic extract.

Sl. No	RT	Name	Formula	Mass	*m*/*z*
1.	1.276	Anthranilic acid	C_7_H_7_NO_2_	137.047	138.0542
2.	4.407	N-Feruloyltyramine	C_18_H_19_NO_4_	313.1301	314.1373
3.	5.262	Caseadine	C_20_H_23_NO_4_	341.1626	342.17
4.	5.35	Batatasin II	C_16_H_18_O_4_	274.1217	297.111
5.	5.743	Calophyllin B	C_18_H_16_O_4_	296.1043	297.1116
6.	6.536	Daidzein 4’,7-diglucoside	C_27_H_30_O_14_	578.1667	577.1585
7.	6.993	Nummularine F	C_23_H_32_N_4_O_4_	428.2422	487.2561
8.	7.145	Syringaresinol O-beta-D-glucoside	C_28_H_36_O_13_	580.2226	579.2149
9.	7.609	Berbamine	C_37_H_40_N_2_O_6_	08.2887	607.2821
10.	8.41	Epi-Tulipinolide diepoxide	C_17_H_22_O_6_	322.1413	345.1305
11.	10.222	1-Dehydro-[6]gingerdione	C_17_H_22_O_4_	290.1506	313.1399
12.	14.692	(S)-Nerolidol 3-O-[a-L-Rhamnopyranosyl-(1->4)-a-L-rhamnopyranosyl-(1->2)-b-Dglucopyranoside]	C_33_H_56_O_14_	676.3747	14.692
13.	19.037	Ursolic acid	C_30_H_48_O_3_	456.3656	455.3584
14.	21.156	Ganosporelactone A	C_30_H_40_O_7_	512.2793	535.2688
15.	21.499	Omega-hydroxy behenic	C_22_H_44_O_3_	356.3335	355.3262

**Table 2:** Half maximum inhibition concentration of the methanol extract of *T. sivarajanii* leaves in various antioxidant assays.

Assay	IC_50_/EC_50_ Value (µg/mL)	
	*T. sivarajanii*	Standard (Ascorbic Acid)
DPPH	184.5 ± 2.4	65 ± 1.3
ABTS	24.15 ± 0.13	2.1 ± 0.03
FRAP	4.94 ± 0.32	0.93 ± 0.04

## Data Availability

The original contributions presented in this study are included in the article/Supplementary Materials. Further inquiries can be directed to the corresponding author.

## Supplementary Materials

The following supporting information can be downloaded at https://www.mdpi.com/article/10.3390/ph18121794/s1, Supplementary Figure S1: Biologically safer concentrations of *T. sivarajanii* leaves extract on RAW 264.7 cells using MTT assay; Supplementary Table S1: Percentage change in the levels of cytokines and NO production in *T. sivarajanii* leaf extract with respect to the LPS alone-treated macrophages.

## Abbreviations

The following abbreviations are used in this manuscript:

TSL	Thottea sivarajanii leaf methanol extract
DPPH	2,2-Diphenyl-1-picrylhydrazyl
ABTS	2,2′-azino-bis-(3-ethylbenzothiazoline-6-sulfonic acid)
FRAP	Ferric reducing antioxidant power
LPS	Lipopolysaccharide
BER	Berbamine
NO	Nitric oxide
LC	Liquid chromatography
SOD	Superoxide dismutase
