# Supplementary material for: Phytochemical Screening by HRLC–MS/MS (Q-TOF) and Antioxidant and Anti-Inflammatory Properties of Thottea sivarajanii Leaf Extract
Source: Pharmaceuticals (Basel). 2025 Nov 25;18(12):1794. doi: 10.3390/ph18121794 (PMC12735574; doi:10.3390/ph18121794)
Supplement: Supplementary file 1 [file pharmaceuticals-18-01794-s001.zip › pharmaceuticals-3944983-supplementary.pdf]

pharmaceuticals

MDPI

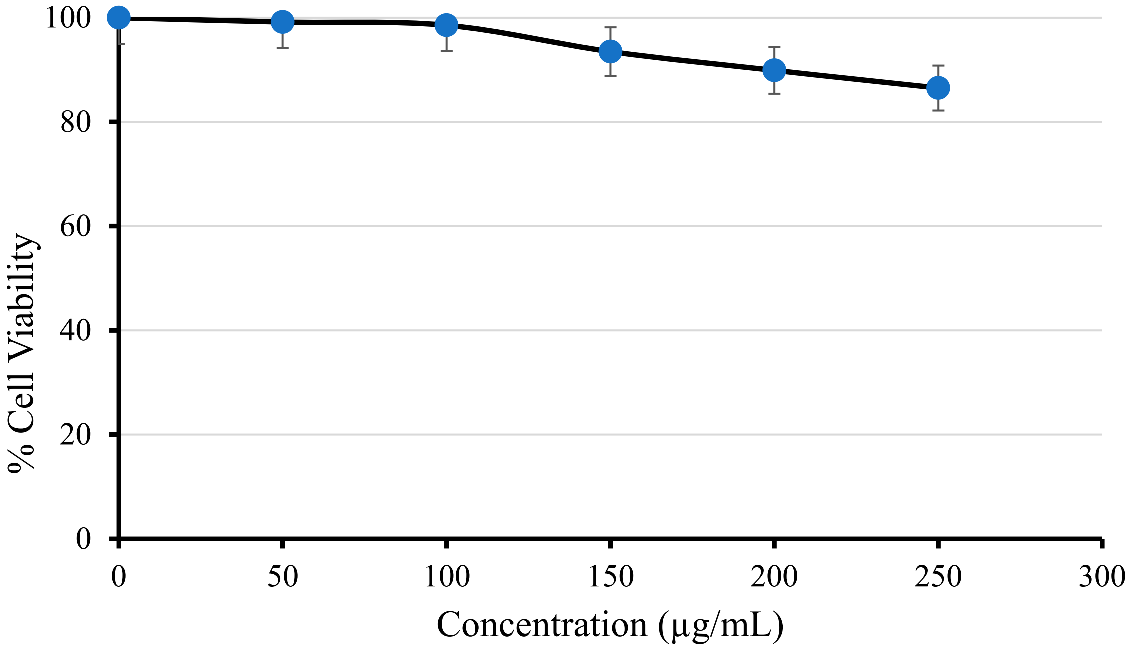

Figure S1. Biologically safer concentrations of *T. sivarajanii* leaves extract on RAW 264.7 cells using MTT assay.

Table S1. Percentage change in the levels of cytokines and NO production in *T. sivarajanii* leaf extract with respect to the LPS alone-treated macrophages .

***T. Sivarajanii* concentration (µg/mL)**	**IL-1β**	**IL-6**	**TNF-α**	**NO**
1.0	37.8± 0.1	32.04± 0.1	17.53± 0.1	28.45± 0.1
5.0	56.0± 0.2	47.43± 0.2	35.90± 0.2	44.31± 0.2
10.0	74.8± 0.2	72.27± 0.2	49.17± 0.2	66.30± 0.2
